# Bending Behaviour of Polymeric Materials Used on Biomechanics Orthodontic Appliances

**DOI:** 10.3390/ma13235579

**Published:** 2020-12-07

**Authors:** Ivo Domagała, Krzysztof Przystupa, Marcel Firlej, Daniel Pieniak, Agata Niewczas, Barbara Biedziak

**Affiliations:** 1 Department of Facial Malformations, Poznan University of Medical Sciences, Bukowska 70, 60-812 Poznań, Poland; ivo.m.domagala@gmail.com (I.D.); marcel-firlej@wp.pl (M.F.); biedziak@ump.edu.pl (B.B.); 2 Department of Automation, Lublin University of Technology, Nadbystrzycka 36, 20-618 Lublin, Poland; 3 Department of Mechanics and Machine Building, University of Economics and Innovations in Lublin, Projektowa 4, 20-209 Lublin, Poland; daniel.pieniak@wsei.lublin.pl; 4 Department of Conservative Dentistry with Endodontics, Medical University of Lublin, Karmelicka 7, 20-080 Lublin, Poland; agata.niewczas@umlub.pl

**Keywords:** orthodontic appliances, bending strength, creep, PMMA

## Abstract

This paper discusses the issues of strength and creep of polymeric materials used in orthodontic appliances. Orthodontic biomechanics is focused on the movement of individual teeth or dental groups as a result of the force applied by orthodontic appliances. Stresses in the construction of functional and biomechanical appliances is generated when using the apparatus in the oral cavity. The orthodontic appliance must maintain its shape and not be damaged during treatment so strength and creep resistance are fundamental properties. It was assumed that the clinical success of orthodontic appliances can be determined by these performance properties. The aim of the work was the experimental assessment of comparative bending strength and creep resistance of selected popular polymer materials used in the production of biomechanical orthodontic appliances. Four commercial materials manufactured by the world class producers were tested: NextDent Ortho Rigid (Vertex-Dental B.V., Soesterberg, The Netherlands) marked as “1A”; Erkocryl (ERKODENT Erich Kopp GmbH, Pfalzgrafenweiler, Germany)-“2A”; Vertex Orthoplast (Vertex Dental B.V.), blue, marked as “3A” and material with the same name as “3A” but orange, marked in the article as “4A”. All the tests were carried out after aging in artificial saliva for 48 h at a temperature of 37 °C. Flexular strength and flexular modulus were made using the three point bending method according to the ISO 178 technical standard. Creep tests were carried out according to the method contained in ISO 899-2. The creep test was carried out in an artificial saliva bath at 37 °C. The creep tests showed significant differences in the strength, modulus and deformability of the tested materials. The strength reliability of the tested materials also varied. The research shows that the 2A material can be used for orthodontic applications in which long-term stresses should be lower than 20 MPa.

## 1. Introduction

The functional properties of polymeric materials, including mechanical properties and their experimental assessment have become an important area of research in the field of material engineering [1,2,3,4]. The growing interest is due to the increasing use of polymers in industrial production as well as medical devices [5,6,7,8,9]. Poly(methyl methacrylate) (PMMA) has been widely used in different fields of healthcare. It is used in orthopedics, prosthodontic dentistry and for many other medical devices [10,11]. The polymer PMMA is one of the most popular thermoplastics due to its physical and mechanical properties: low affectation by ultraviolet radiation, low elongation at break, highly scratch resistant, low moisture and water absorbing capacity, good dimensional stability, high Young’s modulus and hardness, and high volume/weight ratio [12].

Orthodontic biomechanics is focused on the movement of individual teeth or dental groups due to the force applied by orthodontic appliances, selected, fixed and activated by an orthodontist. The orthodontic forcing of a tooth shift is the result of applying active forces to the tooth. The magnitude of tooth displacement depends on the strength and direction of its action as well as root length and alveolar bone height which are the factors determining the locations of the tooth resistance centre and rotation centre. Teeth, together with their supporting structures, react to these forces with a complex biological reaction, which ultimately leads to displacement of teeth in the supporting bone [13].

One of the appliances commonly used in the early development period is the Schwarz plate. The basis of its construction is a lingual or palatal plate. Mechanical elements such as screws, arches and springs are mounted in the plate. The plate design may also contain functional elements that stimulate individual muscle groups to work in the designated direction. These are front or side bite shafts or pelottes. They are made of the same material as orthodontic plates. The material typically used in the production of these elements is PMMA.

The differences are in the production technology of tiles. Traditional methods include the bulk method and the crushed dough method [14]. Both of them are based on making a powder (polymer) and liquid (monomer) by a dental technician. The difference between them is the stage of polymerization the apparatus is formed. As the names of these methods sugest, the apparatus is formed at the dough stage in the crushed dough method and at an earlier stage in the bulk dough method.

**Figure 1:**
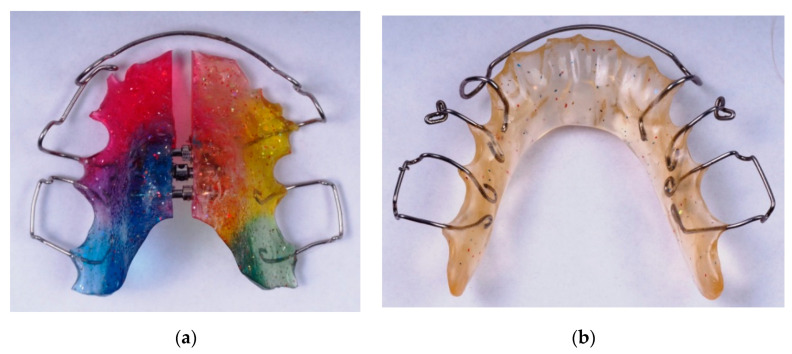
Examples of biomechanical orthodontic appliances: (**a**) upper Shwarz apliance, (**b**) lower Shwarz apliance.

Traditional methods of manufacturing orthodontic appliances are currently accompanied by more modern ones such as the thermoforming method. It involves embossing the shape of the future apparatus plate from a prefabricated thermoformable plate on the plaster model of the patient’s jaw. Plates are available in different thicknesses and colours. Currently, the latest technology is a 3D digital printing of orthodontic appliances designed using appropriate programmes. This method is gaining more and more supporters with the development of digital dentistry, and intraoral scans are gradually replacing traditional dental impressions. Examples of orthodontic appliances manufactured on the basis of orthodontic plates are presented in Figure 1.

Orthodontic appliances are subjected to various biomechanical loads in the oral cavity. Hence, orthodontic panels should be resistant to mechanical deformation or degradation due to environmental factors prevailing in the mouth. Depending on the planned therapeutic effect, the plate is exposed to various forces of different directions and vectors. In addition to para-functions or short-circuit disturbances as well as mechanical volumetric stress, contact forces leading to abrasion also occur.

The issue of reliability of biomedical materials is widely discussed in scientific works. Reliability can be understood as the ability to perform intended functions in relation to time in certain conditions of use [14,15,16,17,18,19]. In this approach, reliability is associated with durability which is also the subject of biomedical materials testing [6,7,20,21,22,23,24,25,26,27,28]. Reliability of mechanical strength is understood differently [29,30,31,32] than reliability referred to as failure rate during use. According to the definition in [33], “The reliability of an object or element is the ability to transfer loads under specific conditions and over a given period of time while maintaining the required strength”. Strength reliability at work has been described in a similar way [34]. The strength of a material is a measure of its resistance to destruction. One of the most important factors affecting strength is the size and distribution of random performance deviations of biomedical products [35]. The weakest link hypothesis assumes that the destruction of a material will occur when a performance deviation/defect reaches or exceeds its critical dimensions. It is assumed that the probability of destruction equals the probability of occurrence of a critical defect [36]. Defect dimensions that determine whether a given defect is critical depend on the stress [37]. For low stresses, only large defects can be critical, while for high stresses, even relatively small defects can become critical [38]. Adopting the weakest link hypothesis allows the use of general parametric equations in modelling the probability distribution. This allows for a general assessment of the biomedical product based on the characteristic probability of destruction in a given sample. This is particularly important for structures that potentially have a dispersion of mechanical strength values [39]. Orthodontic plates can be made manually by a dental technician, so it can be assumed that besides material defects, e.g., air bubbles, plates made of acrylic resins may have manufacturing deviations determining the strength of the material. Therefore, one of the aims of the work was to assess the strength reliability of orthodontic plates used to manufacture orthodontic appliances.

Even in the case of mobile orthodontic appliances, their use is usually associated with the incidence of long-term loads, because the clinical effect of static forces acting for a long time is more favourable [40]. Biomechanical loads occurring during the use of orthodontic appliances which are made of orthodontic plates are static loads. These are biomechanical static monotonic loads that reach the state of stability after a short time after placing orthodontic appliances in the patient’s mouth. Long-term permanent loads in the range below the material elastic limit may lead to material deformation [41]. This process is known as creep [42]. Creep susceptibility to polymeric materials is a subject of scientific research because creep is an immanent feature of many polymeric materials [43,44]. Currently, polymeric materials based on acrylic resins (PMMA) are widely used in the construction of mobile orthodontic appliances and other dental devices [11,45,46,47]. It is expected that materials used for orthodontic products should have durability and dimensional stability during their clinical life.

The second objective of this study is the creep deformation behaviour of four commercial orthodontic plates made of PMMA. Both objectives of the work combine into a superior goal related to the issue of functional properties of technology-dependent orthodontic tile materials from which orthodontic devices are shaped.

## 2. Materials and Research Method

### 2.1. Materials

Four commercial materials were used in the study. NextDent Ortho Rigid (Vertex-Dental B.V., Soesterberg, The Netherlands), based on acrylic resins, marked as “1A”, is a biocompatible material developed for the production of orthodontic elements in the 3D printing technology. The Erkocryl material (ERKODENT Erich Kopp GmbH, Pfalzgrafenweiler, Germany) is an acrylic plastic marked as 2A. The material 2A is intended for the production of orthodontic components. Vertex Orthoplast (Vertex Dental B.V.) is a polymer material based on acrylic resins, intended for the production of orthodontic appliances. This material is suitable for the bulk and crushed dough techniques [14]. Vertex Orthoplast, the plastic 3A, is made transparent and available in 18 colours. This material in the research was in two different colours—dark blue (marked as 3A) and orange (marked as 4A) [15]. The specimens were aging for 48 h in an artificial saliva bath at 37 ± 1 °C in the Q-Cell temperature chamber (Pol-Lab, Wilkowice, Poland).

### 2.2. Specimens Formulation

**Figure 2:**
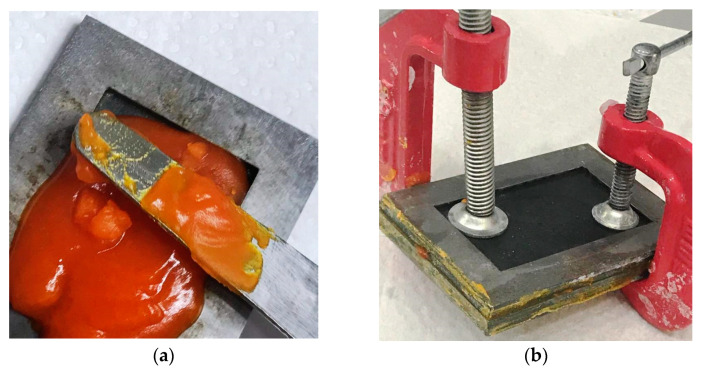

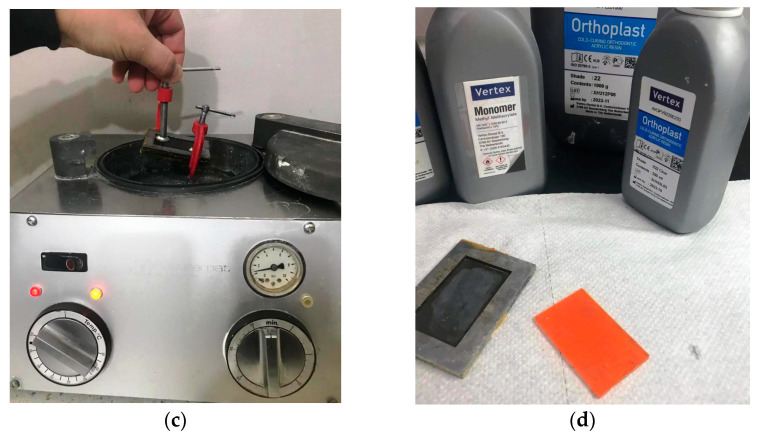
Preparation of the orthodontic plates by the methods of crushed dough and thermoforming: (**a**) applying a flow material 4A to a metal mold, (**b**) forming plates in a metal form, (**c**) plates polymerization equipment, (**d**) hardened orthodontic plate of 4A material after polymerization.

**Figure 3:**
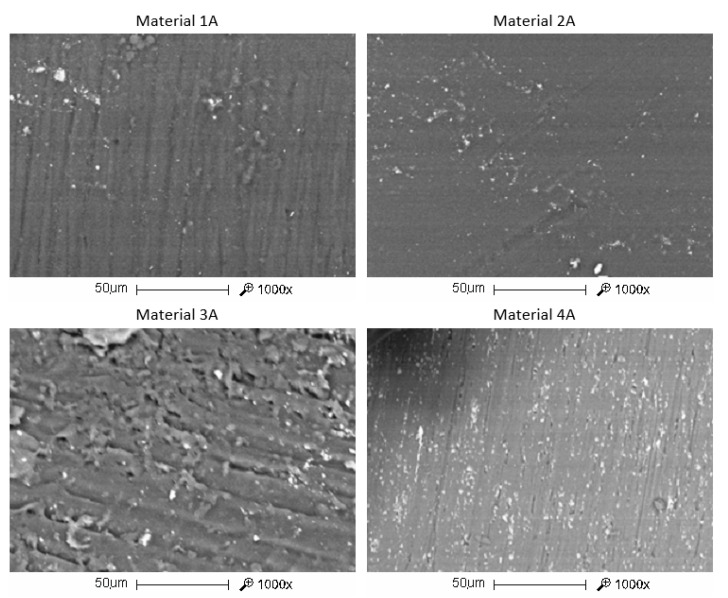
SEM micrographs of the surface of the tested materials (mag. 1000×).

To make the samples, metal dies were cut out with a laser and then glued (Figure 2. The samples made of Vertex Orthoplast (Vertex Dental B.V.) were made using the kneaded dough method. The plates made of the Erkocryl material (ERKODENT Erich Kopp GmbH) were stretched onto the dies in the Erkopress device (ERKODENT Erich Kopp GmbH). A NextDent Ortho Rigid 3D printing was also made from dies which were, however, first scanned and only later printed in a Bego Varseo L printer (BEGO Medical GmbH, Bremen, Germany). The parameters of the tested materials are shown in Table 1. The observations were made with a Quanta FEG 650 (FEI, Hillsboro, OR, USA) scanning electron microscope (SEM). The SEM images of the tested materials are shown in Figure 3.

### 2.3. Flexural Strength and Flexural Modulus

**Figure 4:**
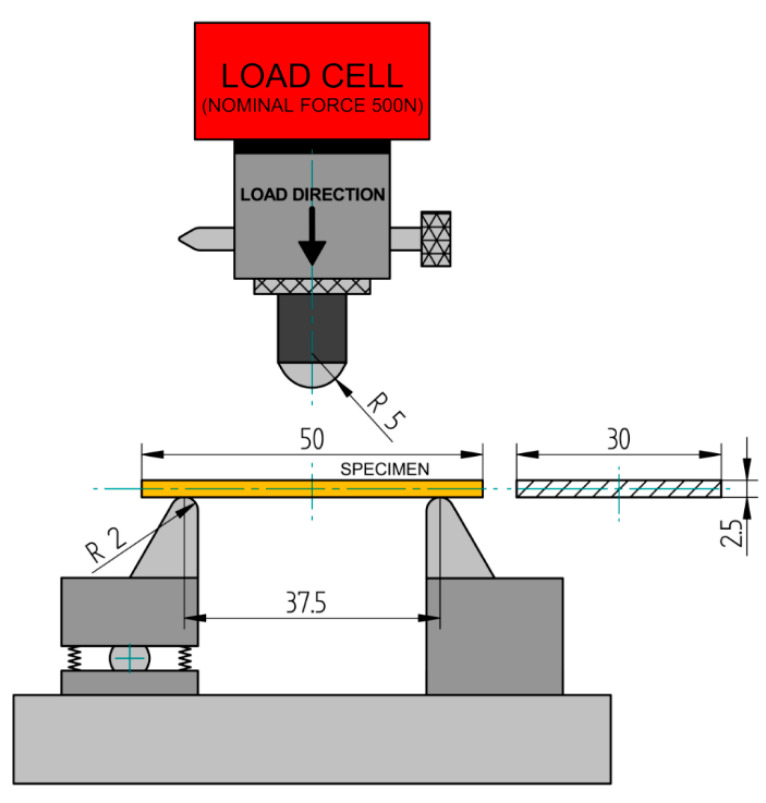
Test system used in the three-point bending test according to ISO 178.

In this work, the bending strength test consisted of conducting a three-point bending test which is experimental modelling [48]. The test is carried out according to a codified method included in the ISO 178 technical standard [49]. The samples used in the study were made as cuboid orthodontic plates (OP) with nominal dimensions of: width *b* = 30 mm, thickness *d* = 2.5 mm, and length *l* = 50 mm. 15 samples of each material were made. The thickness (height) and width of the specimens were measured with a dial caliper. The specimens were aging in an artificial saliva bath at 37 ± 1 °C. The strength test was carried out with a Z100 universal testing machine (Zwick/Roell, Ulm, Germany). The traverse travel speed was 1 mm/min (according to the ISO 178 technical standard) and the support spacing was *L* = 37.5 mm, which resulted from the relationship *L =* (16 ± 1) · *d* according to ISO 178. The radiuses of supports and thrusts as well as other dimensions are given in Figure 4. The test used an Xforce force measuring head with a nominal range of 500 N. The number of samples was assumed to be n = 15, which was based on the work of other authors [50].

The strength (*σ*) was calculated from the following formula:

(1) $$\sigma =\frac{3PL}{2bd^{2}}$$

where P—load during the test [N], *L*—support span [mm], *b*—sample width [mm], and *d*—sample thickness [mm].

The modulus of elasticity characterising the material’s ability of elastic-unstable deformations, was calculated, on the other hand, from the following formula:

(2) $$E_{Y}=\left(\frac{P}{y}\right)\left(\frac{L^{3}}{4bd^{2}}\right)$$

where *P*—load during the test [N], *L*—spacing of supports [mm], *b*—sample width [mm], *d*—sample thickness [mm] and *y*—beam deflection [mm].

### 2.4. Creep Test

**Figure 5:**
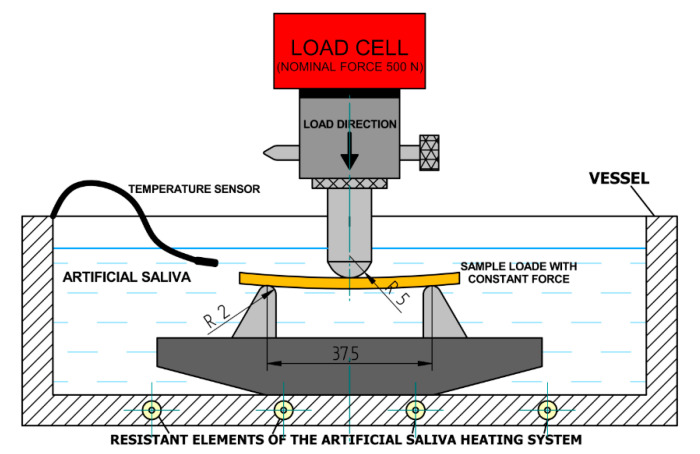
Diagram of the creep test.

The creep tests were carried out according to the method specified in the ISO 899-2: 2005 technical standard titled “Plastics-Determination of creep characteristics-Part 2: Creep when bending under a three-point load” [51]. The creep tests were conducted in medium reflecting real physiological conditions, i.e., in artificial saliva. The composition of artificial saliva has been prepared on the basis of the PN-EN ISO 10271: 2012 standard [52], and the same composition was also used in some other studies [53,54]. The temperature of artificial saliva in the tests was 37 ± 1 °C, which also resulted from the possibilities of the heating system of the measuring vessel. Figure 5 shows the scheme of the research system used in the creep test.

**Figure 6:**
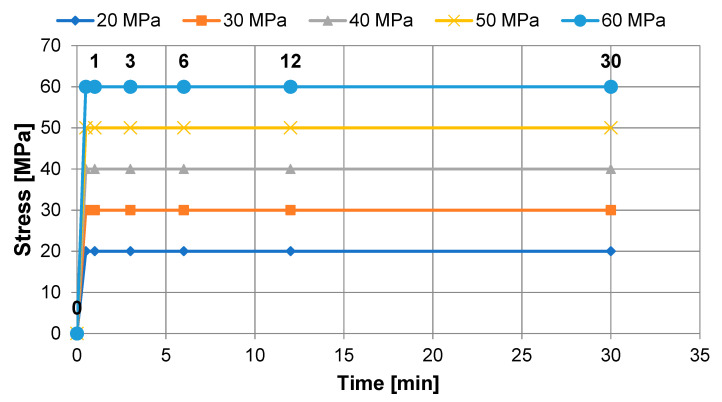
Theoretical stress characteristics-time assumed in the creep test.

The load in the creep test was applied at a speed of 10 mm/min until the threshold stress was reached, which was kept constant until the end of the test (Figure 6). The threshold stress levels were determined in relation to the results of the quasi-static bending test, according to ISO 178, i.e., they were based on the results of the immediate strength measured earlier, which was determined, as described in Section 2.3.

The criteria for ending the creep test were also adopted. The test ended when the maximum deflection of 5% was achieved, and the sample was destroyed when the force dropped by 30%, compared to the maximum force or after reaching the time limit (30 min) if no other test completion criterion was previously achieved.

Creep elasticity modulus was measured in the determined time intervals of the test, i.e., 1, 3, 6, 12, 30 min (Figure 6). The end-of-load creep module over time intervals was calculated as follows:

(3) $$E_{p}=\left(\frac{P}{y}\right)\left(\frac{L^{3}\cdot P}{4bd^{3}y}\right)$$

where *P*—load during the test at the end of the time interval [N], *L*—spacing of supports [mm], *b*—sample width [mm], *d*—sample thickness [mm] and *y*—beam deflection at the end of the time interval [mm].

### 2.5. Statystical Analysis

The results were submitted to the Shapiro-Wilk test of normality, analysis of variance (ANOVA), and multiple comparisons were carried out using Tukey’s HSD post hoc test. The analyses were performed at a 0.05 level of significance. The two-parameter Weibull distribution was used to analyse strength reliability. Weibull Analysis was performed according to the method presented in [36]. One of the major factors influencing strength is the size and distribution of random manufacturing deviations [33]. Due to statistical scattering, the results were subjected to Weibull Analysis to determine the equivalent statistic strength (*σ_e_*) of OP, equal to scale coefficient from the Weibull distribution. The scale parameter understood as specific strength corresponds to 63.2% of cases of damage of orthodontic plates (OP). Additionally, the Weibull modulus (*m*), i.e., shape distribution parameter was determined. The Weibull modulus can be considered as a uniqueness strength parameter (dispersion) of OP. The procedure of Weibull Analysis was described in [36,38].

The modulus of elasticity obtained in the creep test correlation was calculated. The correlation coefficient determines the degree of correlation between the values of two variables [55]. The Pearson linear correlation coefficient was determined [56]. It is worth noting that the Pearson correlation does not depend on the units of measurement of the analysed variables [56]. The correlation coefficient values are in the range from 0 to 1. The highest value of the coefficient *r* means that the correlation is the highest [56].

## 3. Results and Discussion

### 3.1. Flexurar Strength and Elastic Module

**Figure 7:**
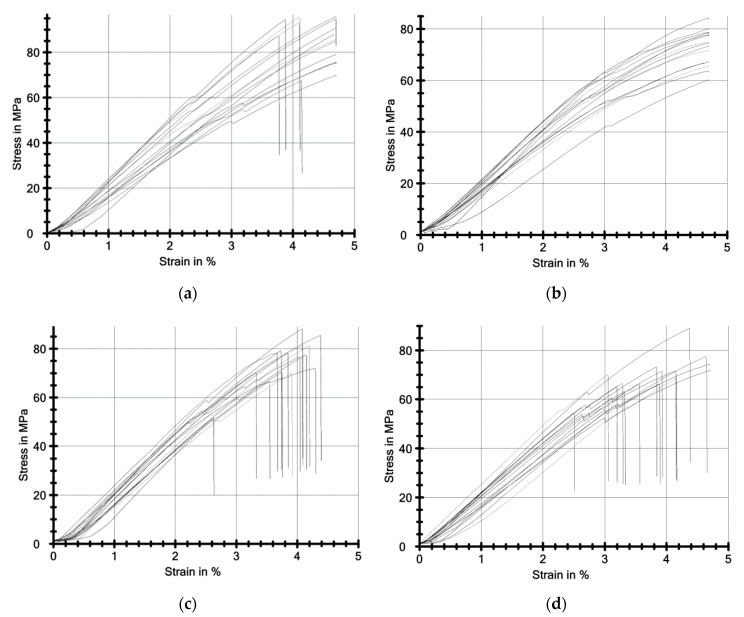
Graphs from the three-point bending test (stress-strain). (**a**) Material 1A, (**b**) Material 2A, (**c**) Material 3A, (**d**) Material 4A.

Figure 7 presents the stress-strain curves (sample deflection) from the three-point bending test. Stress is expressed in mega pascals (MPa), while strain is expressed as a percentage (%).

Table 2 presents the results of the three-point bending test. The following values were presented: n-size of the tested group, E_F_-modulus of elasticity, *σ_fC_*-standard stress, with a sample deflection equal to 1.5 times of the thickness *d* of the tested sample, *σ_fM_*-maximum bending stress (bending strength), *σ_fM_*-deflection corresponding to *σ_fM_*, *σ_fB_*-stress at the time of destruction of the sample, *ε_fB_*-deflection corresponding to *σ_fB_*, *σ_ε=4.7%_*-stress at the maximum allowable deflection of the sample for materials that do not show *ε_fM_* and do not undergo maximum deformation (4.7%), $\bar{x}\;$-average, s-standard deviation, ν-coefficient of variation, L^q^_5%_-bottom 5th percentile of distribution.

Among the tested materials, the material 1A showed the highest average bending strength. The lowest average bending strength was obtained for the material 4A. The highest average value of the flexural modulus was characterised by the material 3A. The material 2A had the lowest modulus of elasticity and the lowest minimum modulus of elasticity (Table 2). The highest statistical dispersion of the modulus of elasticity, expressed by the coefficient of variation ν, was obtained for the material 1A (Table 2) and the lowest for the material 3A.

To measure the parameters of the representative distribution of the normal distribution for the population, i.e., the probability that the sample comes from a population with a normal distribution, the Shapiro-Wilk test was performed. The value of the Shapiro-Wilk (W) statistics was calculated to determine the compliance of the test results with the normal distribution. In the W test, the following assumptions were made: significance level α = 0.05, the null hypothesis *H*_0_: the test results have a normal distribution (for *p* > α) and the alternative hypothesis *H*_1_: the test results have no normal distribution (for *p* < α). Table 3 presents the results of the test for the normality of the statistical distribution of the results of flexural strength tests, and Table 4 of the results of the flexural modulus tests.

The statistical calculations in Table 3 confirm that the results of flexural strength tests similar to those of flexural modulus tests in Table 4 have a normal distribution in all groups. An analysis of variance (ANOVA) was carried out in the next stage of the statistical evaluation. For the results obtained in the bending test, one-way analysis of variance (analysis for one variable) was used for the four groups according to [57]. Levene’s test and the Brown-Forsythe test were used to analyse homogeneity of variance. For the null *H*_0_ hypothesis, the variances in the different groups are homogeneous (for *p* > 0.05) and for the alternative *H_1_* hypothesis, they are heterogeneous (for *p* ≤ 0.05). The results of the tests for homogeneity of variance indicate that the variances in the groups of test results for flexural strength are homogeneous (Levene’s test *p* = 0.5287; Brown-Forsythe test *p = 0.7099).* The results of homogeneity tests on the variance of the flexural modulus indicate that the variances in the groups are heterogeneous (Levene’s test *p* = 0.0042; Brown-Forsythe test *p* = 0.0104). Therefore, variance analysis was performed for the results of flexural strength tests. The null H_0_ hypothesis: the mean values in the groups are the same (for *p* > 0.05) and the alternative *H_1_* hypothesis: at least two mean values differ from each other (for *p* ≤ 0.05) were adopted. The ANOVA test shows that the level of probability *p* between groups for the dependent variable “flexural strength” is *p* < 0.05, which indicates that the null hypothesis should be rejected and the alternative hypothesis should be accepted: at least two average values differ from each other.

The “post hoc” test was carried out to assess differences between groups (materials). Tukey’s HSD test was chosen, based on the analysis of contrasts in groups of measurement results, i.e., honest significant differences in groups (HSD) [56]. The Tukey’s test requirements [57] made it possible to perform an analysis for the results of flexural strength and flexural modulus. The differences between the measurement results are indicated by the significance values of *p* differences. The *p* values below the assumed level (*p* < 0.05) indicate significant differences between the results parameters for the materials. Table 5 presents the results of the Tukey’s HSD “post hoc” test for the variable “flexural strength” and in Table 6 for the variable “flexural modulus”. The values indicating significant differences are marked in red.

### 3.2. Reliability of Strength

**Figure 8:**
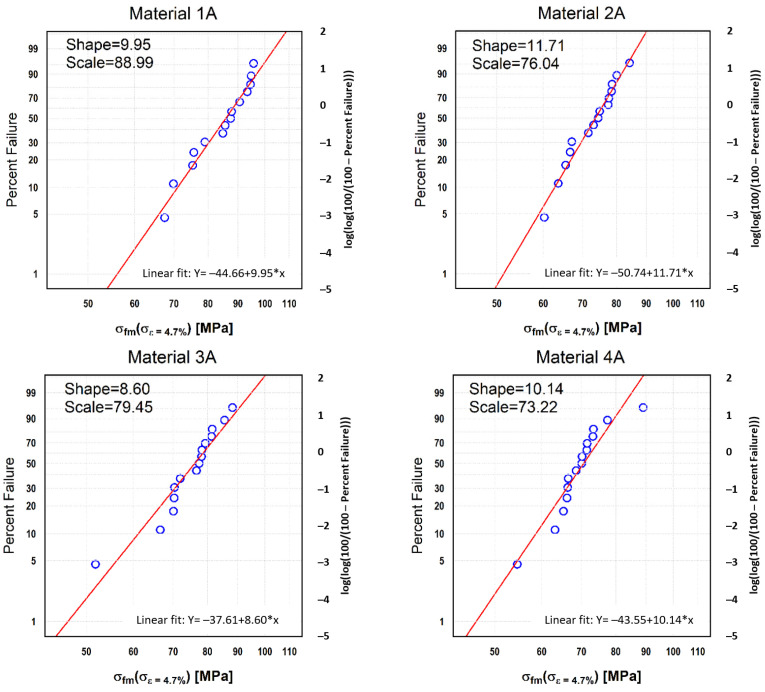
Weibull’s grids describing the flexural strength.

Mechanical strength of orthodontic plates (OP) specifies material behaviour under influence of quasi-static loads. One of the major factors influencing strength of (OP) is the size and distribution of random manufacturing deviations [35], and it is worth mentioning that dental applications are manufactured manually. Due to statistical scattering, the results were subjected to Weibull Analysis. The results of Weibull Analysis of damaging load of orthodontic plates and the approximation of probability of damage are given in Figure 8. The highest value of the Weibull module was obtained for the OPs made of the 2A material (Figure 8). This means that the plates made of this material showed the lowest dispersion of bending strength. The lowest value of the Weibull module was obtained for the tiles made of the 3A material. However, the differences in the value of the Weibull modulus of flexural strength of the tested materials were not very high but significant. The difference between the highest and lowest value was 3.19.

The scale parameter determines the equivalent statistic strength (*σ_e_*) of OP. Its most favourable (highest) value was obtained for the 1A material. Its values were by ~8.5 MPa, ~13 MPa, and ~15.7 MPa higher for the second (3A), third (2A) and fourth (4A) material in the ranking, respectively.

### 3.3. Creep Test Results

**Figure 9:**
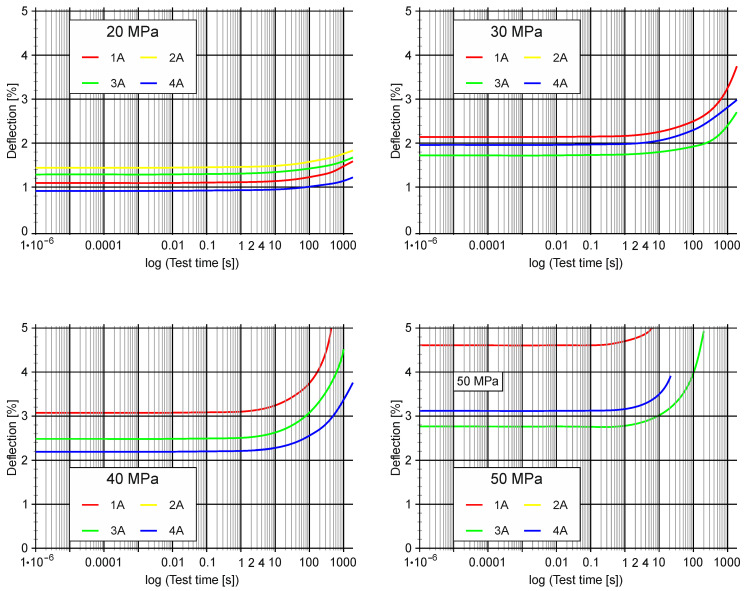
The course of deformation changes over time (logarithmic) during creep.

Figure 9 presents the results of the creep test. These are the graphs of the sample deflections in terms of percentage depending on the test time in logarithmic terms. Table 7 and Table 8 present the results of the creep tests. The following values were presented: *E_t_*—flexural modulus of elasticity at bending, *σ_t_*-stress load of the sample expressed in stress, *ε_t_*—deflection of the sample under load *σ_t_*, *σ_fract_*—breaking stress, *ε_fract_*—deflection of the sample at the time of destruction, *τ_fract_*—time to destruction.

Under the conditions of the creep test, which was carried out in an artificial saliva bath at a temperature of ~37 °C, under 20 MPa, the lowest deformability was observed for the material 4A and the highest for 2A. Under the load which corresponded to a stress of 30 MPa in the sample the material 3A showed the lowest deformation and the material 2A the highest. For the material 2A, the sample was destroyed before reaching 30 MPa. Under the load which corresponded to 40 MPa in the sample, the material 4A had the lowest deformability, whereas for the material 2A the stress of 40 MPa was not achieved in the sample.

Deformability is a quantity related to the stiffness of the material. Another measured quantity that determines the stiffness of a material is the flexural modulus of creep (*E_t_*). The highest values of this parameter were for the 4A material in all load ranges and the lowest ones for the 2A material. The differences are significant because under the load that corresponded to a stress of 20 MPa in the sample, the value of creep (*E_t_*) of the material 2A varied from 1299.98 MPa (Table 7 and Table 8) to 1081.27 MPa and for the material 4A from 1951.37 MPa to 1616.98 MPa (Table 8). The difference in the values (*E_t_*), expressed as a percentage, under load 1, i.e., 20 MPa in the 1st minute of operation was 66.62% and in the 30th minute was 66.87% (Table 7 and Table 8). This means that the variability of the modulus of elasticity, under load 1 was similar for both of the compared materials. Pearson’s correlation analysis of the modulus of elasticity modulus for load 1 (Table 9) indicates that in this respect all of the tested materials behaved similarly. In the subsequent load ranges, the behaviour of the tested materials was more diverse.

The bearing capacity of the orthodontic plates tested was also varied. Under the load that corresponded to 50 MPa in the sample, the material 1A was destroyed after 6.1 s, 2A (no stress reached 50 MPa), 3A after 222.2 s, 4A after 21.7 s of loading. The material 3A showed the highest resistance to the 50 MPa stress cases. Under the load that corresponded to 60 MPa, for the 1A material, the stress of 60 MPa was not reached, 2A (the stress of 60 MPa was not reached), 3A was destroyed after 0.5 s, 4A (the stress of 60 MPa was not reached). The material 3A showed the highest resistance to 60 MPa of stress. It is worth emphasising that only a plate made of the 3A material has withstood the load corresponding to 60 MPa of stress.

## 4. Discussion

PMMA polymers are used to manufacture medical devices that have specific applications and conditions of use [11]. For many PMMA applications, including orthodontic braces made of polymer materials from this group, it is important to know their behaviour under constant biomechanical loads, environmental factors of use, i.e., humidity and temperature. Many polymers soften at elevated temperatures [58]. In [59] it was stated that creep increases at higher temperatures than room temperature and in a humid environment. In this case, it is about elevated temperatures but not the thermal decomposition [60]. According to [61,62], the oral temperature range for men and women is 35.7–37.7 °C and 33.2–38.1 °C, respectively. According to other studies [63], the temperature in the mouth is in the range 36.3–37.1 °C for men, 36.5–37.3 °C for women. PMMA demonstrates increased flexibility in a liquid environment compared to a dry environment, and storage at 37 °C makes PMMA less resistant to fracture than storage at 21 °C [11,63]. Therefore, oral environment factors were included in the experiment. This approach to testing orthodontic appliances seems appropriate because the use of new polymer materials in orthodontic appliances will increase their applicability. Moreover, according to [64,65], a further increase in use is associated with the recognition of the behaviour of a given material in conditions corresponding to real conditions of use.

Bending strength and flexural modulus are important features of medical devices made of PMMA. Many studies have evaluated these properties [11,66,67]. Based on the test results presented in this article, differences in bending strength were demonstrated. The reserve of strength in relation to stresses caused by biomechanical loads affects material efficiency. Also, the mechanism of destruction of the OP in the adopted test conditions was varied. Some samples of the tested materials underwent catastrophic damage. This mechanism of destruction applied to most of the samples made of the materials 3A and 4A (these materials differed only in colour) and some of the samples made of the material 1A (Figure 6). In [68], this problem is explained as follows. Catastrophic damage to polymers is caused by voids and initiation and propagation of brittle cracks. Under these conditions, an uncontrolled crack spread begins in the material, i.e., brittle cracking. The definition of catastrophic damage is presented in [69], and this type of brittle damage of PMMA based materials is illustrated in [70]. For the 2A material and some samples of the 1A material, a plastic destruction mechanism was observed. Ductile polymers fail by crazing or matrix shear yielding. Both mechanisms lead to high crack initiation energy [68].

The OP materials strength and modulus are very important for clinical success. This success depends on the generation of appropriate forces by the orthodontic apparatus. The ability to carry such forces is related to the load capacity of OP, which is related to the strength of the material. However, the rigidity of the orthodontic appliance depends on the modulus of elasticity of the material [71]. The results of orthodontic plate (OP) tests indicate variability of these properties. Weibull analysis makes it possible to address the problem of property differentiation. The value of the Weibull modulus is associated with the standard deviation of strength and modulus of elasticity. The high value of the Weibull modulus indicates the repeatability of strength and modulus of elasticity [38,72]. In [73] it was shown that the standard deviation of strength can affect the decrease in the ability to transfer stress by a degree even higher than the effect of changes in the average value [74]. It seems that the differences in the module values and the Weibull scale parameter could have been influenced by the adopted criteria for the destruction of the plates (samples). The failure corresponded to the stress *σ_fM_*, i.e., the value of the stress at the moment of sample rupture or *σ_ε__=4.7%_* (according to ISO 178, it is the stress at the maximum allowable deflection of the sample for materials not showing *σ_fM_* and not subject to destruction in the range to deformation of 4.7%). The value *σ_ε__=4.7%_* was mostly related to the plates made of the 2A material. Bełzowski [75] explains that the deformability of the sample under load is represented by the average state of the material structure (tile), and the bending strength σ_fM_ depends on the damage that causes the greatest local weakness, which in practice relates to technological or manufacturing defects.

PMMA are inherently viscoelastic materials with time dependent mechanical properties, and understanding their creep behaviour is important [76]. The creep of PMMA is a result of long time application of stress. This problem is of critical importance to evaluate durability and dimensional stability of medical polymer applications [22]. The research presented in this work was carried out for the samples immersed in artificial saliva to simulate the oral environment. In [77] it was found that in the environment of physiological fluids a decrease in polymer creep resistance may occur, which may be associated with the diffusion of liquid particles between polymer chains. On the one hand, these particles can act as a plasticising agent and, on the other hand, as a factor causing stress corrosion, which accelerates the process of material destruction [78,79]. The coexistence of biomechanical forces and oral environment factors for several hours a day determines the durability of the biomechanical orthodontic appliance. Damage in the form of deformation, cracking or loss of stiffness cannot be accepted from a clinical point of view.

## 5. Conclusions

From the results of the research, the following conclusions were drawn:

(1) The mechanical properties of OP depend on the material technology and type of loading.
(2) The efficiency, quality and clinical durability of the orthodontic appliance depend indirectly on the reliability of the properties of materials the apparatus is made from. Despite the fact that polymeric materials for the same purpose but not of the same chemical composition were tested, their reliability varied. It seems that the technology of producing orthodontic plates had an impact. The most reliable (due to its repeatability of strength) material 2A is produced in modern technology. The tiles made of this material were produced using special industrially manufactured moulds and equipment.
(3) Under constant load conditions, which may lead to creep, the tested materials show different strength, stiffness and deformability. Therefore, this property should be taken into account by the orthodontist as the biomechanical effect of the apparatus may be affected.
(4) It seems that among the tested materials, the material 2A, of which orthodontic plates are made, can be used for applications in which stresses caused by long-term biomechanical loads should be less than 20 MPa.

## Figures and Tables

**Table 1:** Specifications of acrylic resin-based materials used in this study.

Material	Batch Number	Expiration Date	Code	Material Composition
OrthoRigid	XK445N01	2019-11	1A	Methacrylic oligomers, phosphine oxides, colorants and pigments
Erkocryl	11198	2022-04	2A	Polymethylmethacryate
Vertex	XH212P05	2023-11	3A	Methyl methacrylate, ethylene glycol dimethacrylate N,N-Dimethyl-*p*-toluidine
Vertex	XH153L03	2023-10	4A	

Domagała 2020.

**Table 2:** Descriptive statistics of the results from the three-point bending test of the tested materials.

Parameter	*E_f_*	*σ_fC_*	*σ_fM_*	*ε_fM_*	*σ_fB_*	*ε_fB_*	*σ* *_ε = 4.7%_*
Unit	MPa	MPa	MPa	%	MPa	%	MPa
Material 1A							
$\bar{x}$	2301.59	81.23	84.33	4.40	88.15	4.11	81.85
s	346.50	8.86	9.97	0.37	10.55	0.32	8.60
ν	15.05	10.90	11.83	8.42	11.97	7.84	10.51
L^q^_5%_	1558.35	61.20	62.36	3.58	61.02	3.28	60.81
n	15.00	10.00	12.00	12.00	6.00	6.00	7.00
Material 2A							
$\bar{x}$	2089.55	55.38	-	-	-	-	72.95
s	277.00	4.26	-	-	-	-	6.92
ν	13.26	7.69	-	-	-	-	9.48
L^q^_5%_	1495.38	46.25	-	-	-	-	58.11
n	15.00	15.00	0	0	0	0	15.00
Material 3A							
$\bar{x}$	2405.11	46.30	75.18	3.84	75.18	3.84	-
s	149.46	5.99	8.88	0.44	8.88	0.44	-
ν	6.21	12.94	11.81	11.58	11.81	11.58	-
n	15.00	3.00	15.00	15.00	15.00	15.00	-
Material 4A							
$\bar{x}$	2129.86	73.98	69.98	3.82	69.51	3.68	74.49
s	199.48	3.43	7.56	0.66	8.04	0.60	2.86
ν	9.37	4.64	10.81	17.25	11.57	16.24	3.84
L^q^_5%_	1701.97	59.20	53.76	2.40	51.99	2.38	62.18

**Table 3:** Shapiro-Wilk test of the W and *p* values of the bending strength test results.

Test	Group (Material)	W	*p*
Bending test	1A	0.91	0.14
	2A	0.96	0.66
	3A	0.92	0.16
	4A	0.92	0.21

**Table 4:** Shapiro-Wilk test of the W and *p* values of the flexural modulus test results.

Test	Group (Material)	W	*p*
Flexural modulus tests	1A	0.93	0.32
	2A	0.97	0.89
	3A	0.97	0.915
	4A	0.97	0.90

**Table 5:** Tukey’s test results.

Tukey’s Test (Bending Test)				
Materials	{1}-M = 84.82	{2}-M = 72.95	{3}-M = 75.18	{4}-M = 69.82
1A {1}		0.00	0.01	0.00
2A {2}	0.00		0.88	0.72
3A {3}	0.01	0.88		0.29
4A {4}	0.00	0.72	0.29	

**Table 6:** Tukey’s test results.

Tukey’s Test (Flexural Modulus Tests)				
Materials	{1}-M = 2301.62	{2}-M = 2089.60	{3}-M = 2405.34	{4}-M = 2129.39
1A {1}		0.11	0.68	0.26
2A {2}	0.11		0.01	0.97
3A {3}	0.68	0.01		0.02
4A {4}	0.26	0.97	0.02	

**Table 7:** Creep test results.

No	Stage Number	Load Time	*E_t_*	*ε_t_*	*σ_t_*	*σ_fract_*	*ε_fract_*	*τ_fract_*
		min	N/mm^2^	%	MPa	MPa	mm	s
Samples 1A								
1	1	1	1658.20	1.21	20	-	-	-
	2	3	1577.14	1.27	20	-	-	-
	3	6	1508.26	1.33	20	-	-	-
	4	12	1426.71	1.40	20	-	-	-
	5	30	1268.94	1.58	20	-	-	-
2	1	1	1242.70	2.41	30	-	-	-
	2	3	1150.35	2.61	30	-	-	-
	3	6	1079.8	2.78	30	-	-	-
	4	12	971.82	3.09	30	-	-	-
	5	30	799.80	3.75	30	-	-	-
3	1	1	1115.97	3.58	40	-	-	-
	2	3	986.20	4.06	40	-	-	-
	3	6	851.80	4.7%	40	-	-	-
4	1	1	-	-	50	50	6.2	6.1
5	-	Sample destroyed before the 60 MPa load level was reached						
Samples 2A								
1	1	1	1299.98	1.54	20	-	-	-
	2	3	1257.30	1.59	20	-	-	-
	3	6	1221.74	1.64	20	-	-	-
	4	12	1166.59	1.71	20	-	-	-
	5	30	1081.27	1.85	20	-	-	-
2	-	Sample destroyed before the 30 MPa load level was reached						
3	-	Sample destroyed before the 40 MPa load level was reached						
4	-	Sample destroyed before the 50 MPa load level was reached						
5	-	Sample destroyed before the 60 MPa load level was reached						

**Table 8:** Creep test results.

No	Stage Number	Load Time	*E_t_*	*ε* *_t_*	*σ* *_t_*	*σ* *_fract_*	*ε* *_fract_*	*τ* *_fract_*
		min	N/mm^2^	%	MPa	MPa	mm	s
Samples 3A								
1	1	1	1417.59	1.41	20	-	-	-
	2	3	1371.97	1.46	20	-	-	-
	3	6	1336.12	1.50	20	-	-	-
	4	12	1281.8	1.56	20	-	-	-
	5	30	1194.99	1.67	20	-	-	-
2	1	1	1586.02	1.89	30	-	-	-
	2	3	1507.86	1.99	30	-	-	-
	3	6	1437.73	2.09	30	-	-	-
	4	12	1331.72	2.25	30	-	-	-
	5	30	1110.92	2.70	30			
3	1	1	1368.34	2.92	40	-	-	-
	2	3	1221.08	3.28	40	-	-	-
	3	6	1112.35	3.60	40	-	-	-
	4	12	977.47	4.09	40	39.90	5.40	1043.70
4	1	1	1403.99	3.56	50	-	-	-
	2	3	1094.03	4.57	50	50	6	222.20
5	1	1	-	-	60	59.9	4.8	0.50
Samples 4A								
1	1	1	1951.37	1.02	20	-	-	-
	2	3	1883.68	1.06	20	-	-	-
	3	6	1829.03	1.09	20	-	-	-
	4	12	1752.68	1.14	20	-	-	-
	5	30	1616.98	1.24	20	-	-	-
2	1	1	1331.30	2.25	30	-	-	-
	2	3	1236.50	2.43	30	-	-	-
	3	6	1167.80	2.57	30	-	-	-
	4	12	1098.01	2.73	30	-	-	-
	5	30	1004.30	2.99	30			
3	1	1	1608.20	2.49	40	-	-	-
	2	3	1482.29	2.70	40	-	-	-
	3	6	1386.11	2.89	40	-	-	-
	4	12	1260.52	3.17	40	-	-	-
4	1	1	-	-	50	49.90	4.80	21.70
5	-	Sample destroyed before the 60 MPa load level was reached						

**Table 9:** Correlations matrix of creep module of elasticity on load 1 (20 MPa of stress).

*r C*oefficient Values*, n = 5*				
Materials	1A	2A	3A	4A
1A	1.00	0.99	0.99	0.99
2A	0.99	1.00	0.99	0.99
3A	0.99	0.99	1.00	0.99
4A	0.99	0.99	0.99	1.00

## References

[1] Lejeune J., Le Houérou V., Chatel T., Pelletier H., Gauthier C., Mülhaupt R. (2018). Creep and recovery analysis of polymeric materials during indentation tests. Eur. J. Mech. A/Solids.

[2] Krzyzak A., Kucharczyk W., Gaska J., Szczepaniak R. (2018). Ablative test of composites with epoxy resin and expanded perlite. Compos. Struct..

[3] Dulebová L., Duleba B., Krzyzak A. (2014). Study of the mechanical properties re-processed of thermoplastic polymer composites. Adv. Mater. Res..

[4] Sun S., Przystupa K., Wei M., Yu H., Ye Z., Kochan O. (2020). Fast bearing fault diagnosis of rolling element using Lévy Moth-Flame optimization algorithm and Naive Bayes. Eksploat. Niezawodn. Maint. Reliab..

[5] Kardach H., Biedziak B., Olszewska A., Golusińska-Kardach E., Sokalski J. (2017). The mechanical strength of orthodontic elastomeric memory chains and plastic chains: An in vitro study. Adv. Clin. Exp. Med..

[6] Pieniak D., Przystupa K., Walczak A., Niewczas A.M., Krzyżak A., Bartnik G., Gil L., Lonkwic P. (2019). Hydro-thermal fatigue of polymer matrix composite biomaterials. Materials.

[7] Pieniak D., Walczak A., Niewczas A.M., Przystupa K. (2019). The effect of thermocycling on surface layer properties of light cured polymer matrix ceramic composites (PMCCs) used in sliding friction pair. Materials.

[8] Pieniak D., Walczak A., Walczak M., Przystupa K., Niewczas A.M. (2020). Hardness and wear resistance of dental biomedical nanomaterials in a humid environment with non-stationary temperatures. Materials.

[9] Ryniewicz A.M., Machniewicz T., Ryniewicz W., Bojko Ł. (2018). Strength tests of the polymers used in dental prosthetics. Arch. Mech. Eng..

[10] Leggat P.A., Smith D.R., Kedjarune U. (2009). Surgical applications of methyl methacrylate: A review of toxicity. Arch. Environ. Occup. Health.

[11] Münker T., Van De Vijfeijken S., Mulder C., Vespasiano V., Becking A., Kleverlaan C., Dubois L., Karssemakers L., Milstein D., Depauw P. (2018). Effects of sterilization on the mechanical properties of poly(methyl methacrylate) based personalized medical devices. J. Mech. Behav. Biomed. Mater..

[12] Almaraz G.D., Martínez A.G., Sánchez R.H., Gómez E.C., Tapia M.G., Juárez J.V. (2017). Ultrasonic fatigue testing on the polymeric material PMMA, used in odontology applications. Procedia Struct. Integr..

[13] Nanda R. (2005). CHAPTER 1—Principles of biomechanics. Biomechanics and Esthetic Strategies in Clinical Orthodontics.

[14] Komorowska A. (2009). Materiały i Techniki Ortodontyczne.

[15] Vertex Orthoplast Shade Guide. https://www.vertex-dental.com/en/products/31-en/26/151-vertex-orthoplast-shade-guide.

[16] Pieniak D., Niewczas A.M., Niewczas A., Bieniaś J. (2011). Analysis of survival probability and reliability of the tooth-composite filling system. Eksploat. Niezawodn. Maint. Reliab..

[17] Bartnik G., Pieniak D., Niewczas A., Marciniak A. (2016). Probabilistic model for flexural strength of dental composites used in modeling reliability of the “tooth-dental composite” system. Eksploat. Niezawodn. Maint. Reliab..

[18] Andrzejczak K., Młyńczak M., Selech J. (2018). Poisson-distributed failures in the predicting of the cost of corrective maintenance. Eksploat. Niezawodn. Maint. Reliab..

[19] Rymarz J., Niewczas A., Pieniak D. (2013). Reliability analysis of the selected brands of city buses at municipal transport company. J. Konbin.

[20] Mahomed A., Jenkins M., Stamboulis A. (2012). Ageing processes of biomedical polymers in the body. Woodhead Publishing Series in Biomaterials, Durability and Reliability of Medical Polymers.

[21] Lewis P.R., Jenkins M., Stamboulis A. (2012). The failure of synthetic polymeric medical devices. Woodhead Publishing Series in Biomaterials, Durability and Reliability of Medical Polymers.

[22] Lewis P.R., Jenkins M., Stamboulis A. (2012). Manufacturing defects in polymeric medical devices. Woodhead Publishing Series in Biomaterials, Durability and Reliability of Medical Polymers.

[23] Pieniak D., Niewczas A.M. (2012). Phenomenological evaluation of fatigue cracking of dental restorations under conditions of cyclic mechanical loads. Acta Bioeng. Biomech..

[24] Łępicka M., Ciszewski A., Golak K., Grądzka-Dahlke M. (2019). A comparative study of friction and wear processes of model metallic biomaterials including registration of friction-induced temperature response of a tribological pair. Materials.

[25] Vasiliu R.-D., Porojan S.D., Birdeanu M., Porojan L. (2020). Effect of thermocycling, surface treatments and microstructure on the optical properties and roughness of CAD-CAM and heat-pressed glass ceramics. Materials.

[26] Pieniak D., Niewczas A., Kordos P. (2012). Influence of thermal fatigue and age-ing on the microhardness of polymer-ceramic composites for biomedical applications. Eksploat. Niezawodn. Maint. Reliab..

[27] Grądzka-Dahlke M. (2010). Analysis of the processes occurring during compression of the porous 316L steel for biomedical applications. Eksploat. Niezawodn. Maint. Reliab..

[28] Żebrowski R., Walczak M., Korga A., Iwan M., Szala M. (2019). Effect of shot peening on the mechanical properties and cytotoxicity behaviour of titanium implants produced by 3D printing technology. J. Health Eng..

[29] Yang C.W., Jiang S.J. (2019). Weibull statistical analysis of strength fluctuation for failure prediction and structural durability of friction stir welded al–cu dissimilar joints correlated to metallurgical bonded characteristics. Materials.

[30] Krzyzak A., Bemowski G., Szczepaniak R., Grzesik N., Gil L. (2018). Evaluation of the reliability of composite materials used in aviation. Safety and Reliability—Safe Societies in a Changing World.

[31] Pieniak D., Ogrodnik P., Oszust M., Niewczas A. (2013). Reliability of the thermal treated timber and wood-based materials in high temperatures. Eksploat. Niezawodn. Maint. Reliab..

[32] Barui S., Panda A.K., Naskar S., Kuppuraj R., Basu S., Basu B. (2019). 3D inkjet printing of biomaterials with strength reliability and cytocompatibility: Quantitative process strategy for Ti-6Al-4V. Biomaterials.

[33] Warszyński M. (1988). Niezawodność w Obliczeniach Konstrukcyjnych.

[34] Quinn J.B., Quinn G.D. (2010). A practical and systematic review of Weibull statistics for reporting strengths of dental materials. Dent. Mater..

[35] Walczak A., Pieniak D., Niewczas A., Niewczas A.M., Kordos P. (2015). Study of ceramic-polymer composites reliability based on the bending strength test. J. Konbin.

[36] Danzer R. (2014). On the relationship between ceramic strength and the requirements for mechanical design. J. Eur. Ceram. Soc..

[37] Todinov M. (2009). Is Weibull distribution the correct model for predicting probability of failure initiated by non-interacting flaws?. Int. J. Solids Struct..

[38] Niewczas A.M., Pieniak D., Ogrodnik P. (2012). Reliability analysis of strength of dental composites subjected to different photopolymerization procedures. Eksploat. Niezawodn. Maint. Reliab..

[39] Babu A.S., Jayabalan V. (2010). Statistical analysis of the fracture strengths of aluminum alloy–alumina (Al_2_O_3_) particulate composites. J. Mater. Sci..

[40] Van Leeuwen E.J., Maltha J.C., Kuijpers-Jagtman A.M. (1999). Tooth movement with light continuous and discontinuous forces in beagle dogs. Eur. J. Oral Sci..

[41] Ferguson S.J., Visser J.M.A., Polikeit A. (2006). The long-term mechanical integrity of non-reinforced PEEK-OPTIMA polymer for demanding spinal applications: Experimental and finite-element analysis. Eur. Spine J..

[42] Spathis G., Kontou E. (2012). Creep failure time prediction of polymers and polymer composites. Compos. Sci. Technol..

[43] Fedorova V.N., Molotkov A.P., Zelenev Y.V. (1973). Investigation of the mechanical properties of acrylics under conditions of creep, stress relaxation, and harmonic vibration. Polym. Mech..

[44] Papanicolaou G., Zaoutsos S. (2019). Viscoelastic constitutive modeling of creep and stress relaxation in polymers and polymer matrix composites. Creep and Fatigue in Polymer Matrix Composites.

[45] Prpic V., Slacanin I., Schauperl Z., Catic A., Dulcic N., Cimic S. (2019). A study of the flexural strength and surface hardness of different materials and technologies for occlusal device fabrication. J. Prosthet. Dent..

[46] Nowakowska-Toporowska A., Malecka K., Raszewski Z., Wieckiewicz W. (2019). Changes in hardness of addition-polymerizing silicone-resilient denture liners after storage in artificial saliva. J. Prosthet. Dent..

[47] Takabayashi Y. (2010). Characteristics of denture thermoplastic resins for non-metal clasp dentures. Dent. Mater. J..

[48] Karbhari V., Strassler H. (2007). Effect of fiber architecture on flexural characteristics and fracture of fiber-reinforced dental composites. Dent. Mater..

[49] PN-EN ISO 178 (2019). Tworzywa Sztuczne—Oznaczanie Właściwości Przy Zginaniu.

[50] Boiko Y.M. (2016). Statistics of strength distribution upon the start of adhesion between glassy polymers. Colloid Polym. Sci..

[51] ISO 899-2 (2005). Plastics—Determination of Creep Characteristics—Part 2: Creep When Bending Under a Three-Point Load.

[52] PN-EN ISO 10271 (2012). Dentistry—Corrosion Test Methods for Metallic Materials.

[53] Walczak M., Pieniak D., Niewczas A.M. (2014). Effect of recasting on the useful properties cocrMoW alloy. Eksploat. Niezawodn. Maint. Reliab..

[54] Manaranche C., Hornberger H. (2007). A proposal for the classification of dental alloys according to their resistance to corrosion. Dent. Mater..

[55] Volk W. (2013). Applied Statistics for Engineers. Literary Licensing.

[56] Bordens K.S. (2002). , Abbott, B.B. Research Design and Methods. A Process Approach.

[57] Hill T., Lewicki P., Lewicki P. (2006). Statistics: Methods and Applications: A Comprehensive Reference for Science, Industry, and Data Mining.

[58] Dmochowska A., Majder-Łopatka M., Salamonowicz Z. (2019). Is it possible to identify a polymer easily. ZN SGSP.

[59] Pieniak D. (2018). Initiation and tolerance of macro-damage of first ply (FBF) in a process of damaging of hybrid multi-ply structures due to reinforcement architecture. Adv. Mater. Sci..

[60] Przybyłek P., Opara T., Kucharczyk W. (2020). comparative Studies of ablative features of the polymer composites used on the thermal shields of Flight data recorders (FDR). ZN SGSP.

[61] Królikowski W. (2012). Polimerowe Kompozyty Konstrukcyjne.

[62] Sund-Levander M., Wahren L.K. (2002). The impact of ADL status, dementia and body mass index on normal body temperature in elderly nursing home residents. Arch. Gerontol. Geriatr..

[63] Hailey J., Turner I., Miles A. (1994). An in vitro study of the effect of environment and storage time on the fracture properties of bone cement. Clin. Mater..

[64] Danel T., Libersa C.Y.T. (2001). The effect of alcohol consumption on the circadian control of human core body temperature is time dependent. Am. J. Physiol. Integr. Comp. Physiol..

[65] Fatemi A., Yang L. (1998). Cumulative fatigue damage and life prediction theories: A survey of the state of the art for homogeneous materials. Int. J. Fatigue.

[66] Komorek A., Komorek Z., Krzyzak A., Przybylek P., Szczepaniak R. (2017). Impact of frequency of load changes in fatigue tests on the temperature of the modified polymer. Int. J. Thermophys..

[67] Lewis G. (2017). Properties of nanofiller-loaded poly (methyl methacrylate) bone cement composites for orthopedic applications: A review. J. Biomed. Mater. Res. Part. B Appl. Biomater..

[68] Slane J., Vivanco J., Meyer J., Ploeg H.L., Squire M. (2014). Modification of acrylic bone cement with mesoporous silica nanoparticles: effects on mechanical, fatigue and absorption properties. J. Mech. Behav. Biomed. Mater..

[69] Bhat R., Mohan N.S., Sharma S., Pratap A., Keni A.P., Sodani D. (2019). Mechanical testing and microstructure characterization of glass fiber reinforced isophthalic polyester composites. J. Mater. Res. Technol..

[70] Chrysafi I., Kontonasaki E., Anastasiou A.D., Patsiaoura D., Papadopoulou L., Vourlias G., Vouvoudi E., Bikiaris D. (2020). Mechanical and thermal properties of PMMA resin composites for interim fixed prostheses reinforced with calcium β-pyrophosphate. J. Mech. Behav. Biomed. Mater..

[71] Perkins W.G. (1999). Polymer toughness and impact resistance. Polym. Eng. Sci..

[72] Sifakakis I., Eliades T. (2017). Laboratory evaluation of orthodontic biomechanics: The clinical applications revisited. Semin. Orthod..

[73] Fairhurst A., Thommen M., Rytka C. (2019). Comparison of short and long term creep testing in high performance polymers. Polym. Test..

[74] Vališ D., Krzyżak A. (2015). Composite materials reliability assessment and comparison. Safety and Reliability of Complex Engineered Systems. Proceedings of the 25th European Safety and Reliability Conference.

[75] Bełzowski A. (2002). Metoda oceny stopnia uszkodzenia kompozytów polimerowych. Kompozyty.

[76] Vaidyanathan T.K., Vaidyanathan J., Cherian Z. (2003). Extended creep behavior of dental composites using time–temperature superposition principle. Dent. Mater..

[77] Chlopek J. (2005). Effects of stress and biological environment on polymeric implants durability. Polimery.

[78] Suwanprateeb J., Tanner K., Turner S., Bonfield W. (1997). Influence of Ringer’s solution on creep resistance of hydroxyapatite reinforced polyethylene composites. J. Mater. Sci. Mater. Electron..

[79] Domagała I., Gil L., Firlej M., Pieniak D., Selech J., Romek D., Biedziak B. (2020). Statistical Comparison of the Hardness and Scratch-Resistance of the PMMA Polymers Used in Orthodontic Appliances. Adv. Sci. Technol. Res. J..

